# Identification and visualization of fusion gene subtypes in APL using spatial attention mechanisms in vision models

**DOI:** 10.3389/fonc.2025.1619296

**Published:** 2025-07-30

**Authors:** Peirou Yan, Guo Pu, Ping Wu, Lijun Wen, Suning Chen, Song Xue

**Affiliations:** ^1^ Department of Hematology, Aerospace Center Hospital, Beijing, China; ^2^ Peking University, Beijing, China; ^3^ Department of Laboratory Medicine, Hebei Yanda Lu Daopei Hospital, Langfang, China; ^4^ National Clinical Research Center for Hematologic Diseases, The First Affiliated Hospital of Soochow University, Jiangsu Institute of Hematology, Institute of Blood and Marrow Transplantation, Collaborative Innovation Center of Hematology, Soochow University, Suzhou, China; ^5^ Hematopoietic Stem Cell Transplantation Department, Beijing Lu Daopei Hospital, Beijing, China

**Keywords:** acute promyelocytic leukemia, pathology, fusion gene subtypes, spatial attention mechanism, bone marrow smear images

## Abstract

**Introduction:**

Acute promyelocytic leukemia (APL) features leukemic cell differentiation arrest at the promyelocytic stage, mainly due to the t (15;17), (q24; q21) translocation that forms the PML-RARA fusion protein. Variant RARα translocations, with distinct biological traits and all-trans retinoic acid (ATRA) responses, often cause misdiagnosis and lengthy genetic testing.

**Methods:**

To solve these problems, we propose a spatial attention mechanism-enhanced convolutional neural network integrating ResNet Blocks and a spatial attention module (CNN with spatial attention), which can achieve high-precision identification of APL fusion gene subtypes and pixel-level visualization of key areas. Data collected from two hospitals and Kaggle, including bone marrow smear images of PML-RARA, TTMV-RARA, NPM1-RARA, STAT5B-RARA, and NUP98-RARG subtypes, were preprocessed to form a five-class dataset.

**Results:**

The model achieves an overall accuracy of 98.04% in five - class classification, with good performance in each category. The attention maps also enhance the model’s interpretability.

**Discussion:**

Such a novel and rapid diagnostic approach for APL subtypes, which achieves high - precision identification and pixel - level visualization, holds significant clinical value.

## Introduction

APL accounts for 10% to 15% of all acute myeloid leukemias (1). The disease is classified under the French-American-British (FAB) classification system as AML-M3. APL is characterized by the blockage of leukemic cells at a specific stage of maturation, namely the promyelocyte stage. The cytogenetic hallmark of APL involves translocation of the RARA gene locus on chromosome 17 (2).A balanced chromosomal translocation (15;17), (q24;q21) between chromosomes 15 and 17 is observed in 95% of cases. This translocation leads to the formation of the PML-RARA fusion protein, which produces a dominant negative mutation that blocks differentiation, while simultaneously preventing apoptosis and enabling the proliferation of leukemic progenitors (3). Multiple variant translocations involving the RARA gene on chromosome 17 have been identified, each characterized by unique rearrangements of the RARα gene paired with various partner genes. These variant translocations are linked to a minority of APL cases, accounting for less than 5% of all APL instances (4).

Approximately 20 mutations have been reported to date, with ongoing discovery of various new variants. Genes involved in APL variants reviewed include:ZBTB16, NPM, NuMA, STAT5B, PRKAR1A, FIP1L1, BCOR, NABP1, TBLR1, GTF2I, IRF2BP2, FNDC3B, ADAMTS17, STAT3, and TFG (5), TTMV (6, 7), NUP98 (8), HNRNPC (9, 10), LBD (11), OBFC2A (12), THRAP3 (13), CPSF6 (14), TBL1XR1 (15). The World Health Organization (WHO) classification system has reclassified these cases as “APL with variant RARA translocation” (16).These cases differ from classical APL in their biological characteristics and response to ATRA (all-trans retinoic acid) dosing, examples include: The ZBTB16-RARA fusion is the result of a reciprocal translocation that involves the ZBTB16 gene located on chromosome11 and the RARA gene situated on chromosome 17 [t(11;17)(q23;q21)].This variant is the most frequently reported and accounts for approximately 1% of all cases of APL (17, 18). Patients with the t(11;17) translocation variant are generally resistant to all-trans retinoic acid (ATRA) (19–22).Since TTMV-RARA APL was first reported by Astolfi et al. in 2021, a total of eight patients have been documented. Among these, only one was an adult, indicating that the condition predominantly affects children or adolescents. In six patients with detailed descriptions of their initial treatment and outcomes, all achieved complete remission (CR) after receiving chemotherapy, either alone or in combination with ATRA and arsenic trioxide (ATO), demonstrating a high remission rate. However, five of these patients relapsed within 1 to 8 months after their initial remission, regardless of whether the consolidation chemotherapy regimen included ATO or ATRA. Additionally, two out of four patients who underwent hematopoietic stem cell transplantation (HSCT) experienced disease recurrence. These unsatisfactory clinical outcomes further emphasize the high malignancy of this APL subtype (6, 7, 23–26).A total of 12 cases of APL with NUP98-RARG/RARA gene rearrangements have been reported. In all treated patients, both ATRA and ATO were ineffective. Eleven patients received induction chemotherapy, and only four achieved CR after the first induction. Ultimately, nine patients achieved CR, with five undergoing HSCT. During follow-up, five patients died, suggesting that the prognosis for APL with NUP98-RARG gene rearrangement may be worse than that of classical APL (8, 27–34).These findings underscore the importance of accurate diagnosis to ensure patients receive appropriate treatment strategies. However, these variants are morphologically similar to classical APL, which can lead to misdiagnosis. Waiting for accurate genetic diagnosis typically takes 1–2 weeks, which has prompted the need for a method to improve diagnostic accuracy based on morphology. The development of deep learning technology is well-suited to meet this demand.

Nicolas Coudray et al. used a CNN to predict mutations in six high-frequency genes (STK11, EGFR, FAT1, SETBP1, KRAS, and TP53) in lung adenocarcinoma (LUAD) from lung cancer tissue section images, with AUC values ranging from 0.733 to 0.856 (35).Riku Nakagaki et al. employed a combination of MaxViT and LightGBM to classify IDH1 mutations in glioma patients, achieving an area under the curve (AUC) of 0.823 for whole-slide images (36). These achievements inspire us to use deep learning to identify different fusion genes. Therefore, our goal is to utilize CNN systems to identify classical APL and its translocation variants through bone marrow smears, and to output their genotypic features.

## Materials and methods

### Data

**Figure 1:**
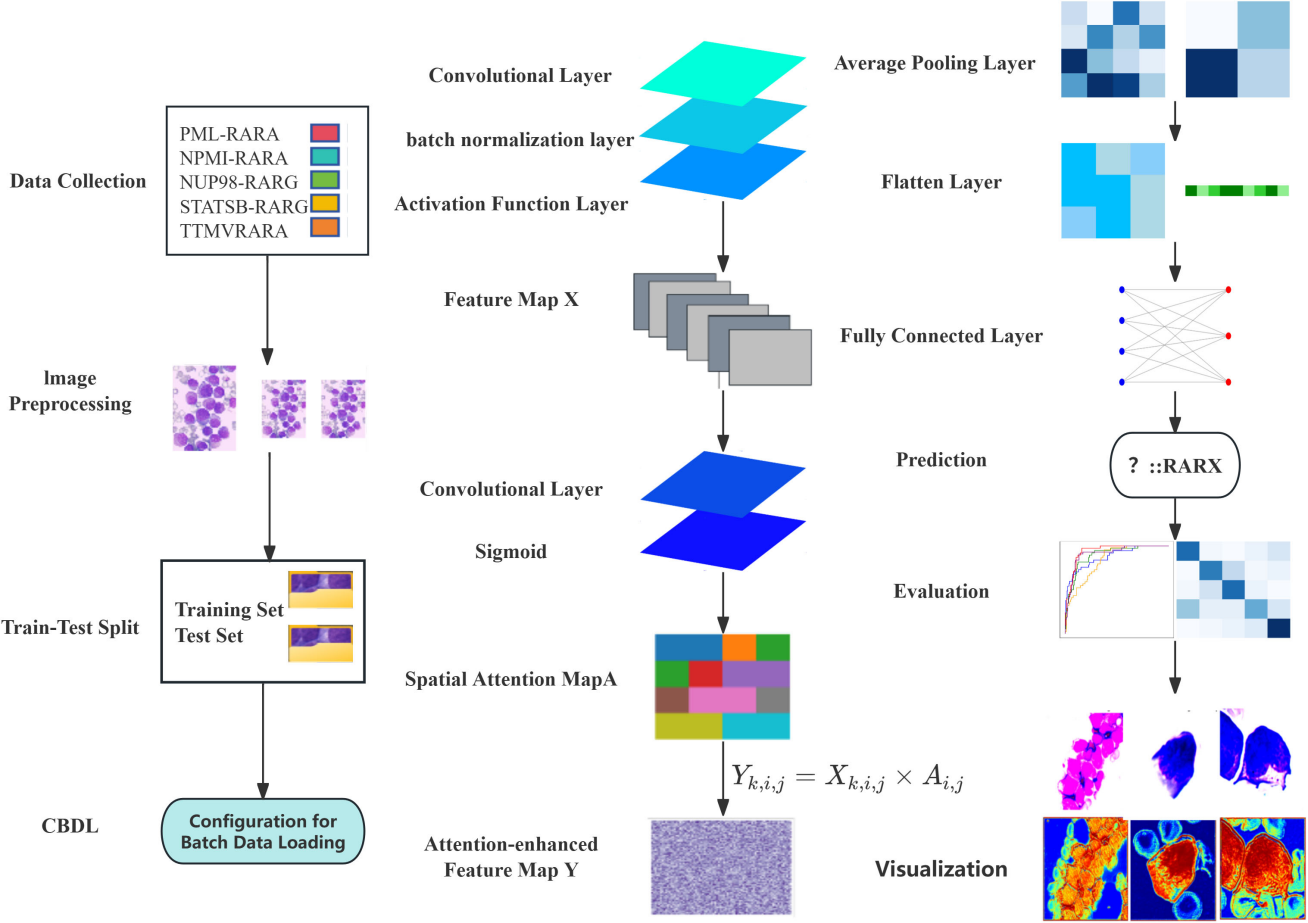
Conceptual framework of spatial attention for bone marrow smear image classification.

Data were sourced from Beijing Lu Daopei Hospital, the First Affiliated Hospital of Soochow University, and the Kaggle database (37). A total of five types of bone marrow smear images were selected, namely PML-RARA, TTMV-RARA, NPM1-RARA, STAT5B-RARA, and NUP98-RARG, with the quantities being 509, 98, 58, 38, and 12 images respectively. (**Figure 1**) Preprocessing included resizing images to a uniform size and normalizing pixel values to stabilize data distribution and facilitate model training. A synthetic dataset with 5 categories was constructed for controlled evaluation.

### Model architecture

#### CNN with spatial attention

In image classification tasks, model interpretability and performance optimization are of utmost importance. Our innovative approach, the Spatial attention mechanism, offers an effective solution to these challenges. The Spatial attention mechanism enables the model to focus on the crucial regions within an image, thereby enhancing the feature representation. Moreover, by visualizing the attention map, we can intuitively understand the model’s decision-making process, identify potential issues, and make targeted improvements.

We constructed a model named CNN with Spatial attention, which combines the ResNet block and the spatial attention module. The input of the model is a bone marrow smear image. The input first passes through a convolutional layer and a batch normalization layer, and then feature extraction is performed through three ResNet block layers. After the first ResNet block layer, a spatial attention module is applied to enhance the feature representation. Next, an average pooling layer is used to down-sample the feature map to reduce its size. Finally, the feature map is flattened and passed through a fully connected layer to output the classification results. During the training process, we used the cross-entropy loss function and the Adam optimizer to optimize the model parameters. To further improve the model performance, a learning rate decay strategy was introduced, which halves the learning rate every certain number of epochs. The cross-entropy loss function is used to measure the difference between the model’s prediction results and the true labels. The Adam optimizer combines the advantages of momentum and adaptive learning rate, which can accelerate the convergence of the model. The learning rate decay strategy gradually reduces the learning rate, enabling the model to converge more stably in the later stage of training. (**Figure 1**)

### ResNet block

To effectively alleviate the common problems of vanishing and exploding gradients in deep neural networks, this model adopts the ResNet block structure. The core idea of the ResNet block is to concatenate the input with the feature after convolution and batch normalization through a shortcut connection, allowing the network to learn the identity mapping more easily. Specifically, each ResNet block consists of two convolutional layers. After each convolutional layer, a batch normalization layer and a Rectified Linear Unit (ReLU) activation function layer are connected in sequence to generate the preliminary Feature map X. The main function of the convolutional layer is to extract local features from the input feature map. Different convolutional kernels can learn various features in the image, such as edges and textures. The batch normalization layer normalizes the input data, making the input data distribution of each neural network layer relatively stable. This not only accelerates the model training process and reduces the problems of vanishing and exploding gradients but also has a certain regularization effect, which helps improve the generalization ability of the model.

The ReLU activation function introduces non-linearity to the neural network. Its formula is f(x) = max (0, x), which can effectively solve the problem of vanishing gradients and has a fast calculation speed, thus being widely used in deep learning. As the core of the ResNet block, the shortcut connection allows the network to directly skip some layers and concatenate the input feature map to the output of subsequent layers, alleviating the gradient problem in deep neural networks and enabling the training of deep neural networks (**Figure 1**).

### Spatial attention module

The Spatial attention module is designed to enhance the model’s focus on important spatial regions within an image. Firstly, the input Feature map *X* is processed through a convolutional layer. This operation results in a single-channel Feature map *M*. Essentially, this process fuses and transforms the information from each channel of the Feature map *X*, extracting preliminary attention information related to spatial positions. The convolutional kernel linearly combines the information from different channels, compressing the multi-channel features into a single channel. As a result, the value at each spatial position comprehensively reflects the feature situation of that position across all channels. (**Figure 1**)

Subsequently, the preliminary attention information *M* is fed into a Sigmoid activation function. This function normalizes the values of *M* to the range of [0,1], generating the Spatial attention map *A*. In this attention map, the value at each position represents the relative importance of that position within the entire feature map. A value closer to 1 indicates that the position is more important and worthy of the model’s attention, while a value closer to 0 implies that the position is relatively less significant.

Finally, the Feature map X is element-wise multiplied with the Spatial attention map A to obtain the final Attention-enhanced Feature map Y. Specifically, for each element $X_{k,i,j}$ in the feature map X (where k is the channel index and i and j are the row and column indices of the spatial position respectively) and the corresponding element $A_{i,j}$ , in the Spatial attention map A, the element $Y_{k,i,j}\;$ in the Feature map Y is calculated through the following element-wise multiplication:


$$Y_{k,i,j}=X_{k,i,j\;}\times A_{i,j}$$

During the multiplication process, the Spatial attention map A acts as a weight. It amplifies the feature responses at important positions (where the attention value is close to 1) and suppresses those at less important positions (where the attention value is close to 0). This enables the model to pay more attention to the important spatial regions in the feature map during subsequent processing, thereby enhancing its ability to capture key information (**Figure 1**).

### Model evaluation

**Figure 2:**
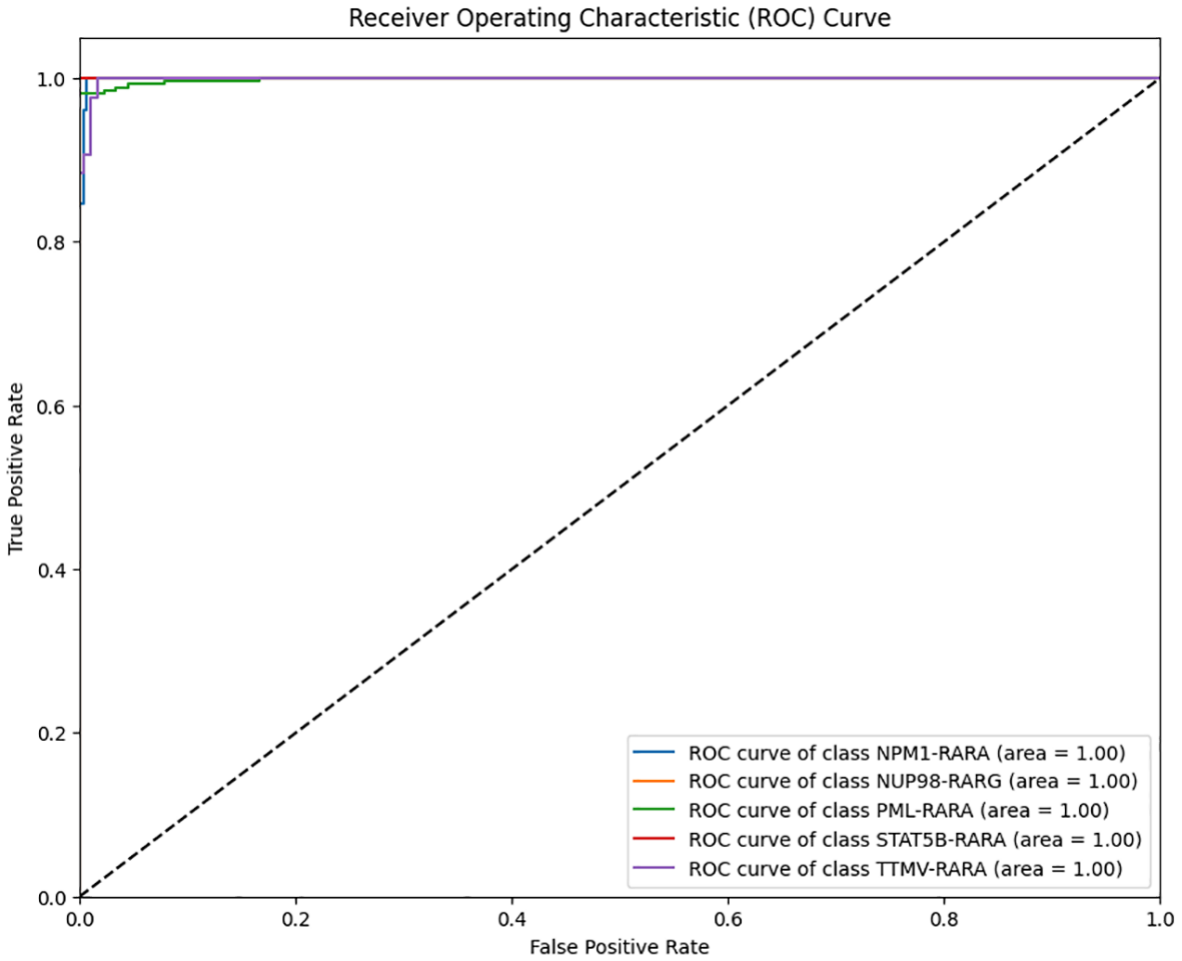
Multi-class Receiver Operating Characteristic (ROC) curve of the classification model on the APL dataset (Blue: ROC curve of class NPM1–RARA; Yellow: ROC curve of class NUP98- RARG; Green: ROC curve of class PML–RARA; Red: ROC curve of class STAT5B–RARA; Purple: ROC curve of class TTMV-RARA.

**Figure 3:**
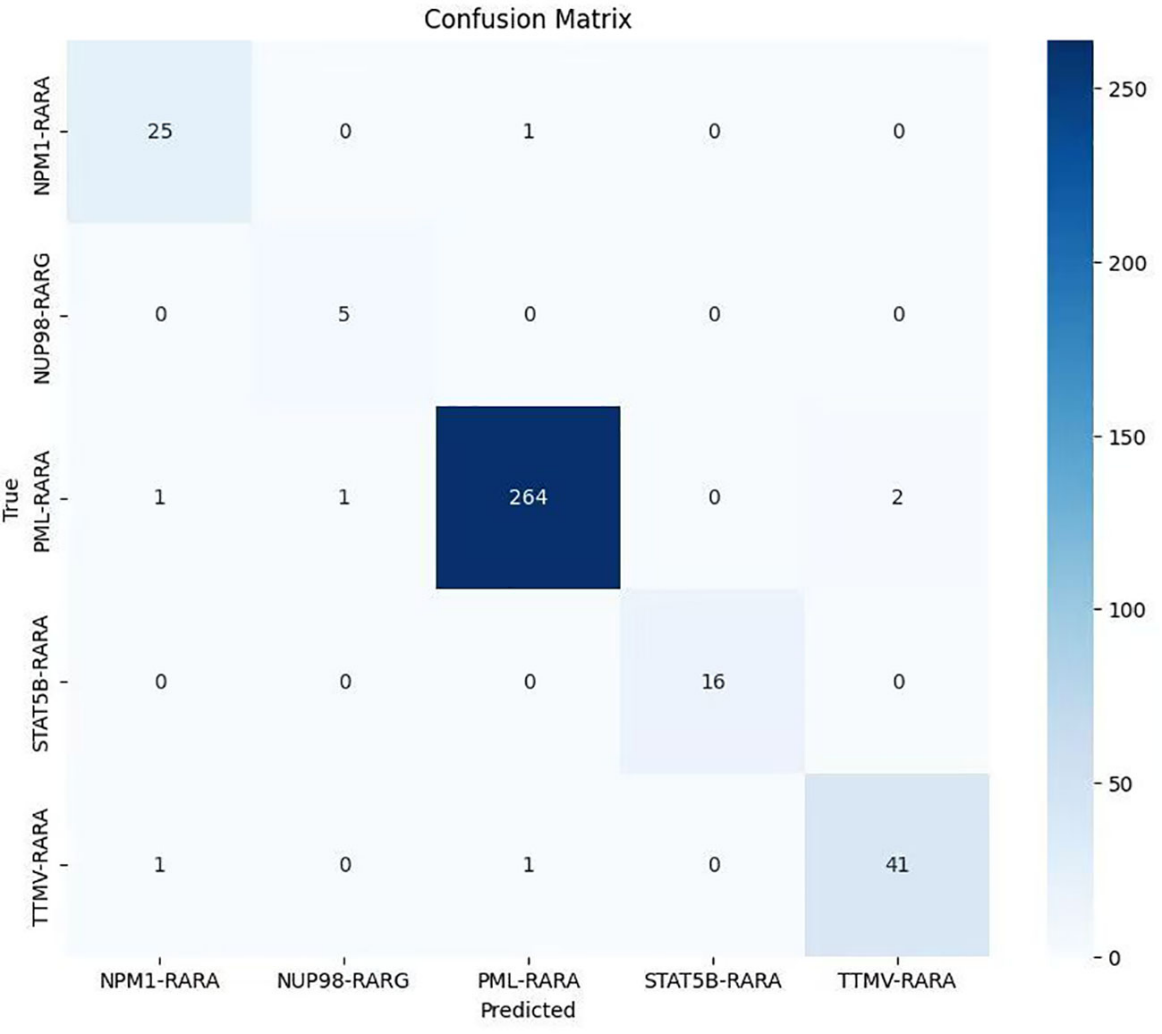
Confusion matrix of the classification model on the APL dataset (The lighter to darker blue hues correspond to the smaller to larger number of samples.

After the model training is completed, the test set is used to comprehensively evaluate the model. The overall accuracy and the accuracy of each category of the model are calculated to intuitively reflect the classification performance of the model. Meanwhile, a confusion matrix and an ROC curve are plotted (**Figures 1**–**3**).

### Visualization of the attention map

To further explore the model’s decision-making, we visualized and overlaid the attention map of a test-set sample on the original image. The map, with colors indicating attention levels, clearly shows the regions the model focuses on. This simple yet effective visualization enhances model interpretability and helps pinpoint areas for model improvement (**Figure 1**).

In the data collection phase, bone marrow smears from patients with PML-RARA, TTMV-RARA, NPM1-RARA, STAT5B-RARA, and NUP98-RARG are gathered and undergo image preprocessing, including resizing, conversion to tensors, and normalization. The dataset is then split into a training set (50%) and a test set (50%) through a train-test splitting. A batch data loading configuration that supports batch processing and random shuffling is employed to load both the training and test sets. Architecturally, the preprocessed images are fed into a convolutional neural network with attention mechanism and residual blocks for the image classification task. First, a Feature map X is generated via a convolutional layer, a batch normalization layer, and an activation function layer. Subsequently, a Spatial attention map A is created using a convolutional layer and a Sigmoid function. Based on the formula $Y_{k,i,j}=X_{k,i,j\;}\times A_{i,j}$ , an attention-enhanced Feature map Y is produced. Then, it passes through an average pooling layer, a flattening layer, and a fully connected layer. The model is trained with the cross-entropy loss function and the Adam optimizer. Finally, prediction results are output, and the model’s performance is evaluated on the test set by calculating the overall accuracy, classification report, confusion matrix, and multi-class ROC curves. For interpretability, one image is randomly selected from each class, and its original image and attention map are visualized to illustrate the regions that the model pays attention to.

## Result

The evaluation of the model on the test set yielded highly satisfactory results. The overall accuracy reached 98.04%, indicating that the model boasts excellent classification capabilities on the whole. It can accurately classify most samples, demonstrating high reliability, generalization ability, and outstanding performance in this classification task. When examining the accuracy of each individual class, the classification performance across different classes was relatively balanced and generally high. Specifically, for the “NPM1-RARA” class, the precision was 0.926 (95%CI: 0.766, 0.979), the recall was 0.962 (95% CI: 0.811, 0.993), and the F1-score was 0.943; for the “PML-RARA” class, the precision was 0.992 (95% CI: 0.973, 0.998), the recall was 0.985 (95% CI: 0.962, 0.994), and the F1-score was 0.989; for the “NUP98-RARG” class, the precision was 0.833 (95% CI: 0.436, 0.970), the recall was 1.000 (95% CI: 0.566, 1.000), and the F1-score was 0.909; for the “STAT5B-RARA” class, both the precision and recall were 1.000 (95% CI: 0.806, 1.000), and the F1-score was 1.000; for the “TTMV-RARA” class, both the precision and recall were 0.953 (95% CI: 0.845, 0.987), and the F1-score was 0.953 (**Table 1**).

**Table 1:** Performance comparison of CNN with a Spatial attention Model on APL Fusion Gene Subtype .

Class	Precision (95%CI)	Recall (95%CI)	F1_Score
NPM1-RARA	0.926(0.766, 0.979)	0.962(0.811, 0.993)	0.943
PML-RARA	0.992 (0.973, 0.998)	0.985(0.962, 0.994)	0.989
NUP98-RARG	0.833 (0.436, 0.970)	1.000 (0.566, 1.000)	0.909
STAT5B-RARA	1.000 (0.806, 1.000)	1.000 (0.806, 1.000)	1.000
TTMV-RARA	0.953 (0.845, 0.987)	0.953 (0.845, 0.987)	0.953
Overall	0.980(0.960,0.990)	NA	NA

The analysis of the multi-class ROC curves indicated that the area under the ROC curve (AUC) for all classes was close to 1. This is an extremely ideal outcome, suggesting that the model achieved the optimal classification performance for each class, which capable of perfectly distinguishing between positive and negative samples with extremely high sensitivity and specificity (**Figure 2**).

The confusion matrix results revealed that most samples were accurately assigned to their corresponding classes, directly indicating the model’s effectiveness in the classification task. The large values on the diagonal of the matrix represent a large number of correctly classified samples, while the relatively small values of the non-diagonal elements indicate that the cases of misclassification were rare. This further validates the model’s excellent performance in distinguishing samples from different classes in the classification task (**Figure 3**).

**Figure 4:**
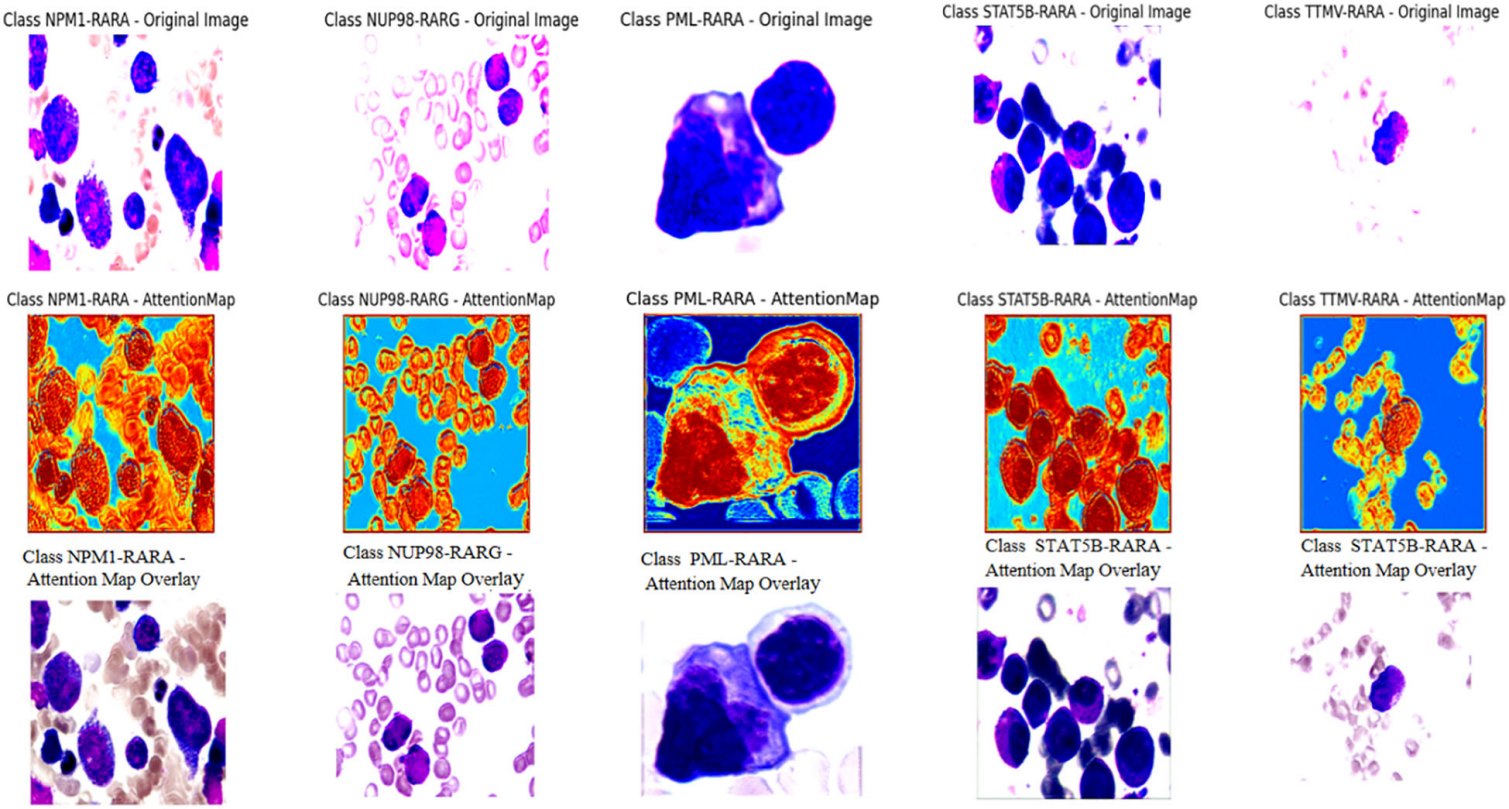
The first row of pictures: Original pathological pictures; The second row of pictures: Visualization of the classification model based on the spatial attention mechanism (in the model’s attention degree, red > yellow > green > cyan > blue); The third row of pictures: Superimposed visualization of the classification model based on the spatial attention mechanism.

The attention mechanism of the model was demonstrated by randomly selecting one sample image from each class for visualization. From the visualization results, it was clear that the model could focus on the key parts of the images. When comparing the original images with the attention maps, the attention maps accurately highlighted the important regions relevant to the classification decision. This indicates that the model’s classification is not a random decision but is based on the effective capture of key image features. Further, by overlaying the attention maps on the original images, the association between the regions the model focused on and the actual content of the images could be more intuitively observed. This not only validates the effectiveness of the model but also provides strong visual evidence for understanding the model’s decision-making process. Overall, the visualization results prove the rationality and accuracy of the model in handling image classification tasks from another perspective (**Figure 4**).

## Discussion

In this study, we innovatively employed a CNN with Spatial Attention to precisely identify classical APL and its translocation variants. The model performed remarkably on the test set, with an overall identification accuracy of 98.04%, showing excellent classification ability. Individual class F1- scores were balanced and high: NPM1-RARA (0.943), PML-RARA (0.989), NUP98-RARG (0.909), STAT5B-RARA (1.000), TTMV-RARA (0.953). Furthermore, the model achieved pixel-level visualization, enabling precise localization of key regions in bone marrow smear images. The Spatial attention mechanism played a pivotal role by assigning different weights to each position in the feature map, adaptively adjusting the focus on regions relevant to the classification task. This mechanism enhanced the feature representation of critical areas, allowing the model to better capture subtle variations in these features while suppressing irrelevant background details. This approach not only improved the model’s adaptability to image variations across different patients, enhancing its generalization ability and reducing the risk of overfitting, but also refined the predictive outcomes of the CNN. The attention maps clearly illustrated the model’s focus areas, with colors ranging from red to blue indicating the intensity of attention from strong to weak. Clinicians can use these maps to further validate the model’s diagnostic results and identify subtle pathological changes that might otherwise be overlooked. For instance, in the attention maps, tumor cells are highlighted with more intense red hues. In the PML-RARA category, the attention maps not only focus on the nucleus but also highlight the location of Auer rods in the cytoplasm. In the STAT5B-RARA category, attention is concentrated at the junction between the nucleus and cytoplasm, potentially reflecting the impact of STAT5B-RARA on the overall cellular state, including signal transduction between the nucleus and cytoplasm. Looking ahead, given that the morphology and distribution of APL cells may change at different stages of the disease, clinicians can analyze the variations in attention maps over time to monitor disease progression, assess treatment efficacy, and adjust therapeutic strategies accordingly.

### Comparison 1: improved pre-trained ResNet50 model

**Figure 5:**
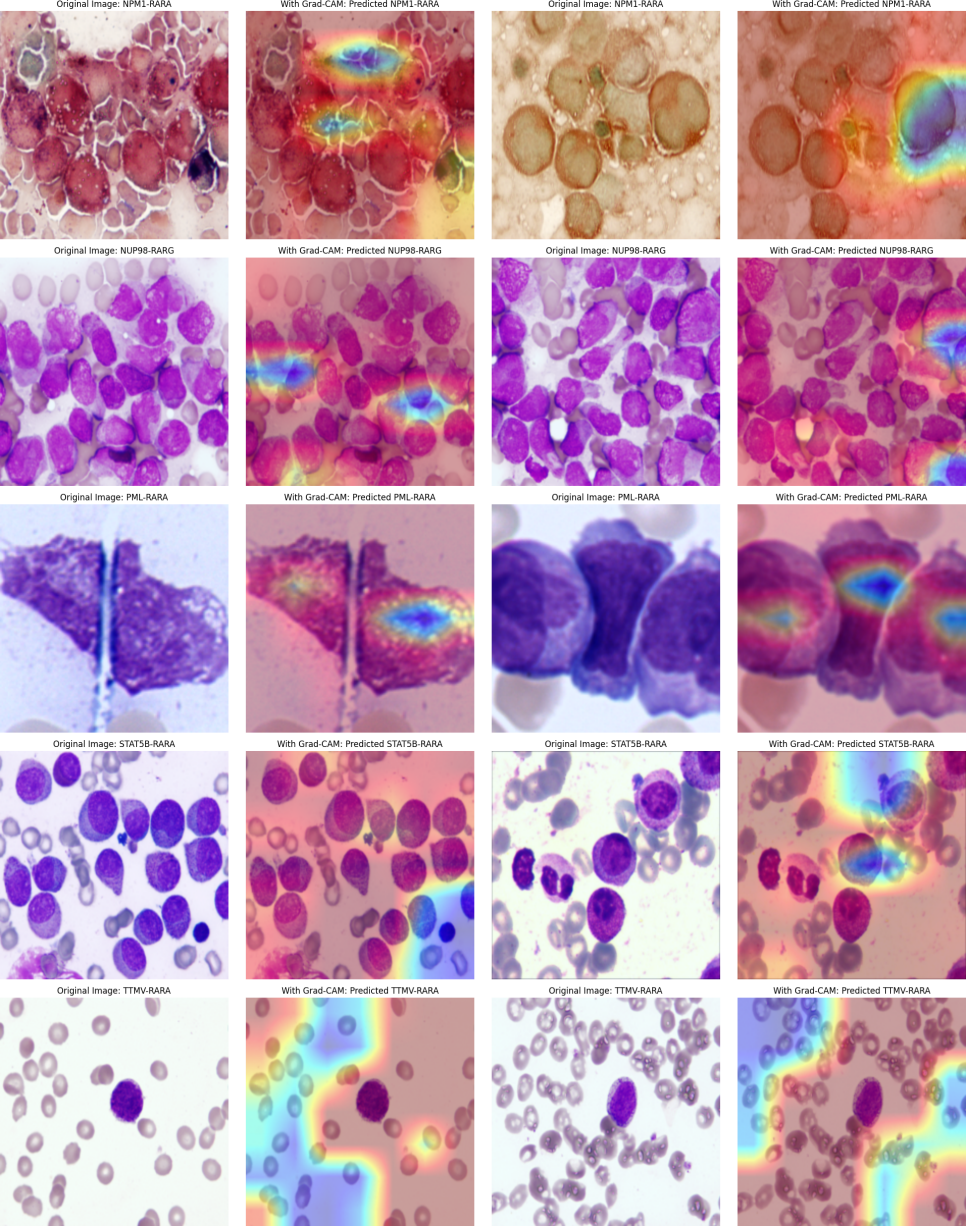
Attention maps of the improved Pre-trained ResNet50 Model: Good accuracy but unstable attention regions.

During the research process, we experimented with various approaches. For instance, we developed a modified version of the pre-trained ResNet50 by inserting a self-attention module before its final convolutional layer. This module calculates attention weights based on queries (Query), keys (Key), and values (Value), generating a weighted feature map that is added to the original feature map as the final output. Additionally, we employed Grad-CAM (Gradient-weighted Class Activation Mapping) to produce heatmaps for visualizing the model’s focus areas in the images. The results showed good overall accuracy 0.958 (95% CI: 0.932,0.974), with F1 accuracies as follows: NPM1-RARA (0.920), PML-RARA (0.978), NUP98-RARG (0.889), STAT5B-RARA (1.000) and TTMV-RARA (0.851). The specific precision, recall and other metrics can be found in **Table 2**. However, the visualization was unstable. During the training process, the visualized regions of interest kept changing, making it difficult to consistently and precisely locate the key regions (see **Figure 5**).

**Table 2:** Performance of improved pre-trained ResNet50 model on APL fusion gene subtype.

Class	Precision (95%CI)	Recall (95%CI)	F1_Score
NPM1-RARA	0.958 (0.798, 0.993)	0.885 (0.710, 0.960)	0.920
PML-RARA	0.974 (0.947, 0.987)	0.981 (0.957, 0.992)	0.978
NUP98-RARG	1.000 (0.510, 1.000)	0.800 (0.376, 0.964)	0.889
STAT5B-RARA	1.000 (0.806, 1.000)	1.000 (0.806, 1.000)	1.000
TTMV-RARA	0.841 (0.706, 0.921)	0.860 (0.727, 0.934)	0.851
Overall	0.958 (0.932, 0.974)	NA	NA

### Comparison 2: multi-layer CNN combined model

**Figure 6:**
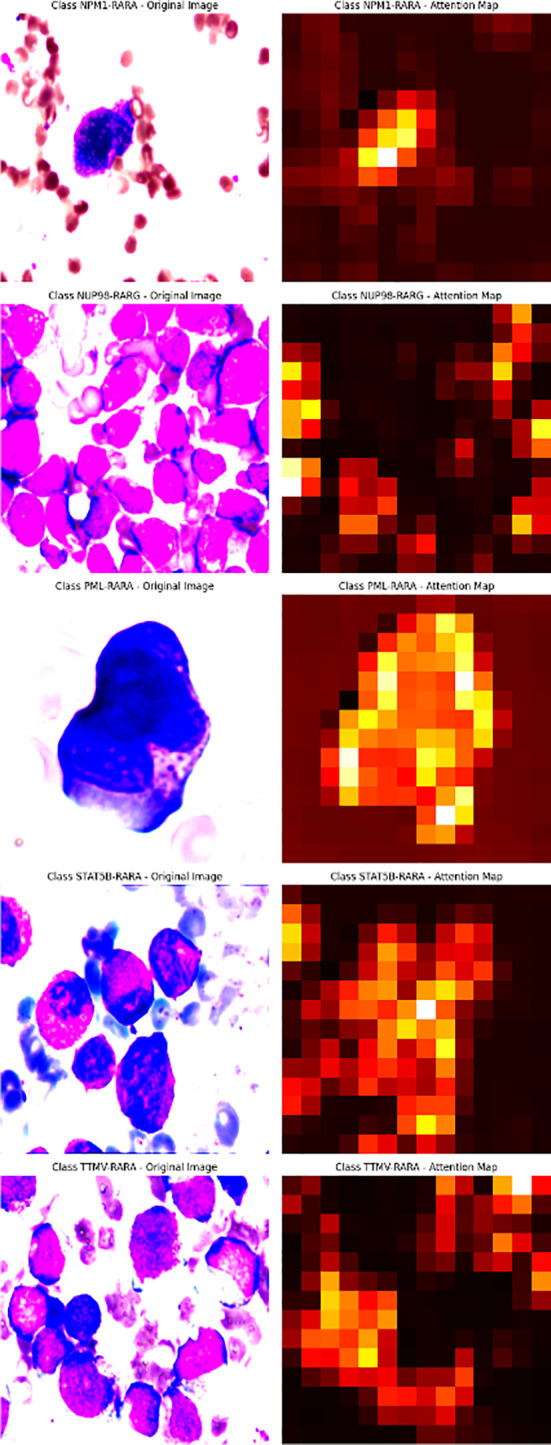
Multi-layer CNN combined model for complex image processing: Blurry Attention Maps.

We further explored a model that combines a convolutional attention mechanism with a multi-layer CNN. The experimental results indicated that this model performed poorly when dealing with complex images, featuring low classification accuracy (the overall accuracy was 0.896, with a 95% CI of 0.860-0.923). The F1 scores for different categories are as follows: NPM1-RARA (0.906), NUP98-RARG (0.000), PML-RARA (0.968), STAT5B-RARA (0.387), and TTMV-RARA (0.733). Specific metrics such as precision and recall can be found in **Table 3**. Moreover, the attention maps were blurred (as shown in **Figure 6**). This suggests that simply combining a basic attention mechanism with a multi-layer CNN did not effectively enhance the model’s feature extraction capabilities. Stacking multiple CNN layers may lead to gradient vanishing or information loss, especially in deeper networks, thus limiting the feature extraction ability.

**Table 3:** Performance of improved multi-layer CNN combined model on APL fusion gene subtype.

Class	Precision(95% CI)	Recall (95%CI)	F1_Score
NPM1-RARA	0.853 (0.699, 0.936)	0.967 (0.833, 0.994)	0.906
PML-RARA	0.977 (0.951, 0.989)	0.958 (0.927, 0.977)	0.968
NUP98-RARG	0.000 (0.000, 0.561)	0.000 (0.000, 0.490)	0.000
STAT5B-RARA	0.462 (0.232, 0.709)	0.333 (0.163, 0.563)	0.387
TTMV-RARA	0.685 (0.553, 0.793)	0.787 (0.651, 0.880)	0.733
Overall	0.896 (0.860, 0.923)	NA	NA

The spatial attention mechanism effectively resolved classification and visualization challenges. However, there is a significant disparity in sample sizes across different subtypes. For rare subtypes like NUP98-RARG, the sample size is extremely limited, with only 12 cases and five images in the test set. This leads to considerable fluctuations in accuracy for such subtypes, in stark contrast to the consistently stable accuracy of the PML-RARA subtype. The imbalance in sample sizes restricts the model’s generalization ability and increases the risk of overfitting. In the future, to augment the data volume for rare subtypes, we will adopt a two-pronged approach. First, we will collect as many bone marrow smear samples as possible. Second, we will apply a series of technical methods to process the existing data. We can perform data augmentation operations on the current images, such as rotation, flipping, scaling, and cropping. Additionally, we will employ oversampling techniques to increase the proportion of rare samples in the training set. Furthermore, we will utilize Generative Adversarial Networks (GANs) or Variational Autoencoders (VAEs) to generate synthetic images that closely resemble real ones.

Our research is pioneering in the field of identifying fusion genes through pathological images, particularly for classical APL and its variants. By leveraging the spatial attention mechanism, we achieved excellent visualization effects and demonstrated the potential of deep learning techniques in advancing pathological slide diagnosis. This study opens new avenues for precise and interpretable AI-driven medical diagnostics.

## Data Availability

The datasets presented in this study can be found in the online Kaggle repository. Specifically, it pertains to the “Human leukemia cytomorphology dataset” accessible at https://www.kaggle.com/datasets. A portion of the data was independently collected. If access is required, one may contact the corresponding author.
